# Supplementary material for: Dynamic transcriptomic profiles of zebrafish gills in response to zinc supplementation
Source: BMC Genomics. 2010 Oct 11;11:553. doi: 10.1186/1471-2164-11-553 (PMC3091702; doi:10.1186/1471-2164-11-553)
Supplement: Additional file 2 — Interactive Direct Interaction Network representing the molecular interactions between zinc, copper, iron, calcium and proteins encoded by transcripts changed by zinc supplementation. Mini web-site containing index.html and hyperlinked pages in subdirectory describing a Direct Interaction Network automatically generated based on curated interactions contained within the proprietary PathwayArchitect database. Ovals represent proteins and the circles symbolize metal ions. Objects are coloured by their abundance in zebrafish at the time-point they were significantly different from the control is a scale from -4 fold (dark green) to +4 fold (dark red). Where significant differences were found at more than one time-point, the colour overlay shows expression at the first instance. Dark blue squares denote 'binding', and light blue squares 'expression'; green squares stand for 'regulation', green diamonds for 'metabolism', and green circles for 'promoter binding'. Arrow heads indicate directionality of the interaction where annotated. All nodes and edges can be further interrogated by selecting the relative area of the image. [file 1471-2164-11-553-S2.zip › PathwayArchitect Zn xs DIN/703166.html]

# REGULATION:

|  |  |
| --- | --- |
| Type | REGULATION |
| Effect | Negative |


---

|  |  |
| --- | --- |
| Score | 0 |


---

|  |  |
| --- | --- |
| Reference Count | 3 |


---

|  |  |
| --- | --- |
| Mechanism | Unknown |


---

|  |  |
| --- | --- |
| Reference:0 || Sentence | "WT1 splice variants lacking a KTS insert between zinc fingers 3 and 4 suppressed IGF-IR promoter activity in the absence of p53 or in the presence of wild-type p53." |
| PMID | 12444079 |
| Year | 2002 |
| Species | Human |
| Journal | J Biol Chem |
| RefScore | 0 |
| Source | PArchNLP |
  |
|  |  |


---

|  |  |
| --- | --- |
 Reference:1 || Sentence | "The purpose of this study was to determine whether the decrease in IGF-IR expression was transcriptionally regulated, and whether WT1 action may be involved in the repression of the IGF-IR gene in prostate cancer cells." |
| PMID | 11145562 |
| Year | 2001 |
| Species | Mouse |
|  | Human |
| Journal | Endocrinology |
| RefScore | 2 |
| Source | PArchNLP |
  ||  |  |


---

|  |  |
| --- | --- |
 Reference:2 || Sentence | "Cotransfection of Chinese hamster ovary cells with rat and human IGF-IR gene promoter constructs driving luciferase reporter genes and with WT1 expression vectors showed that the active WT1 gene product represses IGF-IR promoter activity in a dose-dependent manner." |
| PMID | 8390684 |
| Year | 1993 |
| Species | Human |
|  | Rat |
| Journal | Proc Natl Acad Sci U S A |
| RefScore | 1 |
| Source | PArchNLP |
  |


---

|  |  |
| --- | --- |
